# *Fifty Percent Human* – how art brings us in touch with our microbial cohabitants

**DOI:** 10.1111/1751-7915.13285

**Published:** 2018-06-21

**Authors:** Sonja Bäumel, Hanne L. P. Tytgat, Birgit Nemec, Ruth Schmidt, Loo Wee Chia, Hauke Smidt

**Affiliations:** ^1^ Independent Artist, Designer and Teacher; ^2^ Laboratory of Microbiology Wageningen University & Research Wageningen The Netherlands; ^3^ Institute of Microbiology Swiss Federal Institute of Technology ETH Zurich Zürich Switzerland; ^4^ Department for History and Ethics of Medicine University of Heidelberg Heidelberg Germany; ^5^ Department of Microbial Ecology Netherlands Institute of Ecology Wageningen The Netherlands; ^6^ INRS‐Institut Armand‐Frappier Université du Québec Quebec City QC Canada

## Abstract

The Human Microbiome, as well as the exploration of the microorganisms inhabiting the human body, are not only integral to the field of microbiology but represent an intrinsic part of all human beings. Consequently, along with scientists, artists have been inspired by the microbiome: transforming it in to tangible artefacts in order to critically question, reflect on and break down the barrier between humans and their microcohabitants. By artistic means, artists help us to understand how microbial research topics are inevitably affected by societal influences, including (health) politics, economics and the arts. *Fifty Percent Human* is a multidisciplinary artistic research project that aims to reshape our understanding of the human body and its environment as well as to explore possibilities for conscious coexistence in order to bridge the gap between science and society.

## We are 50% human

With the help of microbiologists, humans are slowly coming to the understanding that our bodies are ecosystems rich in biodiversity, which contain complex societies of microbes living in and on our bodies. One can even ponder how human we really are, when taking into account that microorganisms cover the entire outer surface of our body, including our skin, gut, vagina and mouth (The Human Microbiome Project Consortium, 2012). Drawing on microbiologists’ claims that 90 per cent of cells that constitute our body are not human but bacterial (Savage, 1977; Brown, 2010), we can begin to visualize the human body as a complex ecosystem. Recently, more in‐depth calculations have “upgraded” our humanness, showing that about 50 % of cells being human (Sender *et al*., 2016a,b).

*Fifty Percent Human* was initiated by and is part of Sonja Bäumel's ongoing research and creative process through which a multidisciplinary team examined, questioned and challenged the relationship between the human body and its microbial cohabitants. In particular, the project aimed to see how the concept of the Human Microbiome can be critically challenged and how artists can play a role in translating research, often perceived as something abstract to laymen, into a more tangible and immediate experience. To question and shape the relational and context‐dependent aspects of the human existence, it needs to be grasped as complete as possible, that is down to its tiniest components, including human body cells and the non‐human cohabitants of the body, its microbes.

Today, however, the discrepancy between people's perception of the functions and purposes of microbes and the essentiality of microorganisms to our existence could not be larger. Laymen often perceive microorganisms as dangerous creatures and sources of diseases, taking hygienic practices to the level of germophobia. *Fifty Percent Human* aims to challenge this perception by allowing people to reach out to their microbes and explore the potential of their microbial self. It strives to help people to realize that we are much more than who we think we are and to recognize microbial life forms as actors that coshape our bodies, as well as our physical and mental constitution (Rees *et al*., 2018). If we aspire to truly experience and better understand inter‐organism communication, we may take better care of the microcosm, and ultimately take better care of ourselves (Hird, 2009; Bakke, 2014).

The project started a search for an empathic, sensory and ethical encounter with the microcosm, respecting experiences of difference, strangeness and otherness as a crucial moment in the process of getting to know ourselves. “What would a microbe do?” became the central question spurring this project on (Brand, 2012). This idea was further translated into six questions central to the *Fifty Percent Human* project: (i) Who is there? (i) Where do they come from? (iii) How do they move? (iv) What are they communicating? (v) Who are their companions? (vi) What happens when they meet organisms that are not like them?

## Mapping ‘Microbial Bodies’

Microbes present in and on our body – and by extension in and on other organisms that we are in contact with – are vital to our wellbeing. As this is to a large proportion dependent on how microbes communicate with us and each other, samples were collected from the ‘hands’ of different species. To this end, a specific macrocosm a house in Amsterdam, the Netherlands, was chosen. The microbiota residing on the palms of a human individual's hands, the paws of the individual's cat and on the leaves of a tree in the person's garden were sampled.

**Figure 1:**
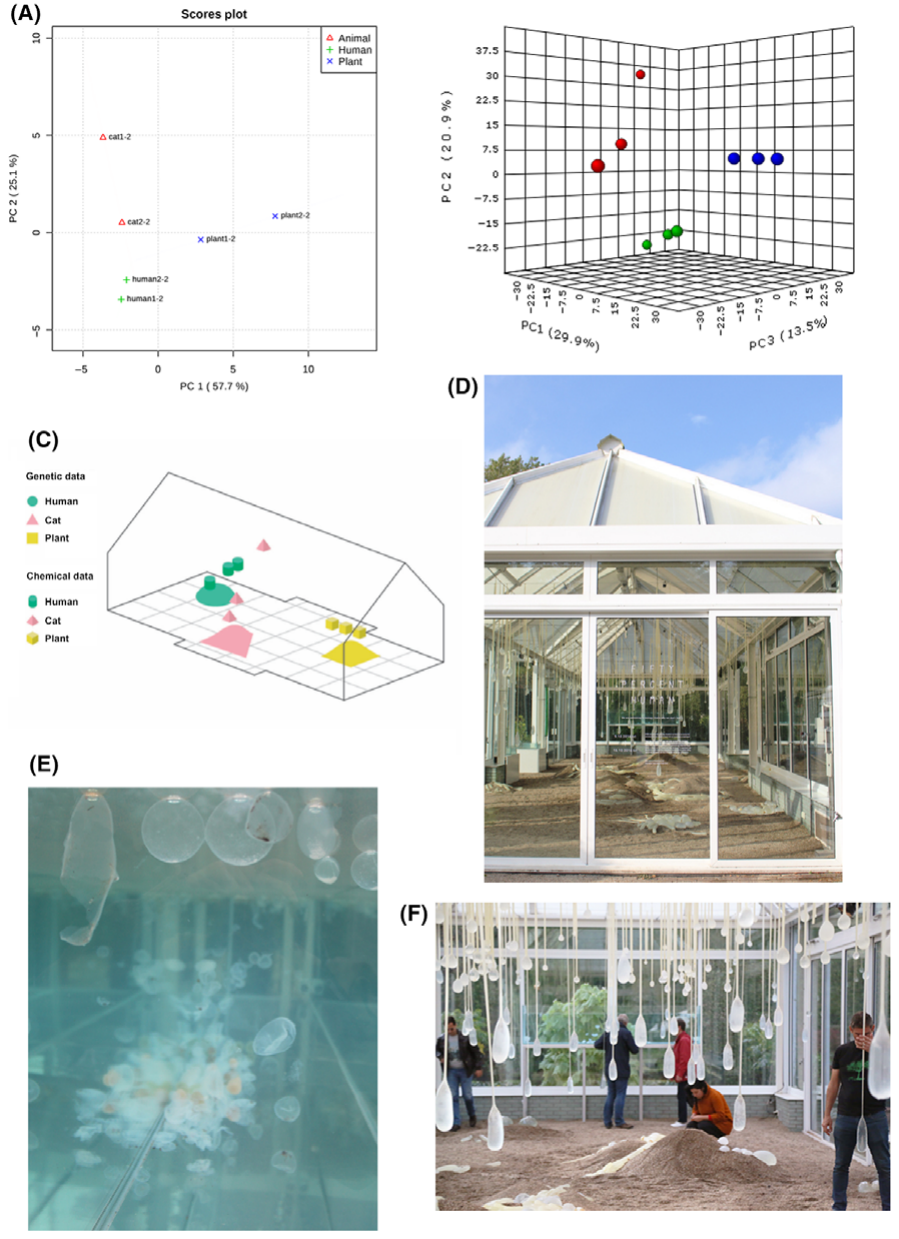
From science to art: scientific data inspired the building of an in‐between space where people could meet their microbes. Principle component analysis (PCA) plots describe the relatedness of the microbial community composition (‘Who is there?’, A) and the relatedness of the volatile organic components (VOC) they emit to communicate (‘What are they communicating?’, B). Red data points represent samples from the cat's paws, green the microbiota of human hands and blue the microbes present on a tree leaf. Three biological replicates are depicted for the VOC analysis, and microbial community analysis is based on two biological replicates for each niche. All PCA plots were generated using MetaboAnalyst (Xia *et al*., 2015). These scientific results were used to inspire the blueprint of the in‐between space, by superimposing both PCA plots graphically (C), and to tangibly materialize (D, E, F) the installation. Panels D, E and F show an impression of the in‐between space as it premiered at Zone2Source, Greenhouse Amstelpark, Amsterdam, the Netherlands in October 2016.

**Figure 2:**
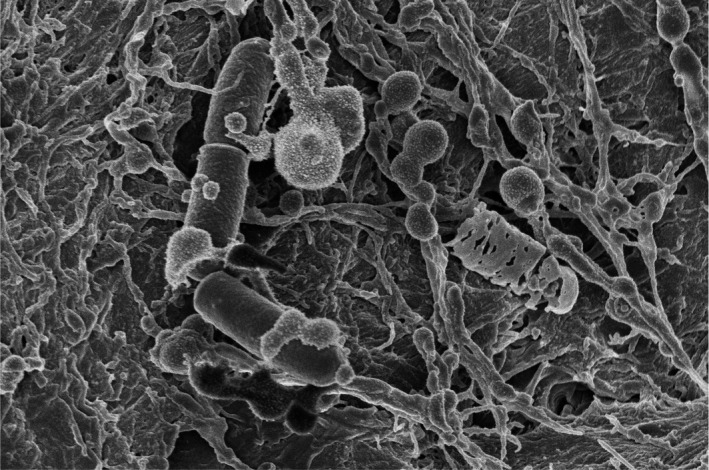
Where are they from? Electron microscopy image from bacteria on human skin.

To answer our first question, that is to find out ‘Who is there?’, a biomolecular analysis of microbial community composition was performed. PCR‐amplified fragments of microbial 16S ribosomal RNA (rRNA) genes of all three samples were analysed using MiSeq Illumina sequencing to explore the archaeal and bacterial content of the samples (Ramiro‐Garcia *et al*., 2016). Similarity of the observed microbial communities was plotted using principal component analysis (PCA) (Fig. 1A). Furthermore, microbial communities were visualized in their original habitat, being on human skin, cat skin and plant leaves using electron microscopy. The electron microscopy also answered the question ‘Where are they from?’ (Fig. 2).

Next to an understanding of ‘Who is there?’, data answering ‘What are they communicating?’ was gathered. Using gas chromatography, volatile organic compounds emitted by the different microbial communities were analysed and compared. PCA analysis was also used here to describe the similarity between the observed microbial communities (Fig. 1B). To explore ‘How do they move?’, ‘Who are their companions?’ and ‘What happens when they meet organisms that are not like them?’, various culturing experiments were set up. The motility of the organisms of each microbiota sample was assessed by following their movement in low melting point agar. By inoculating microbiota samples from each source (i.e. human, tree and cat) on the same plate but in distinct corners, we could follow how they grow towards each other and explore the zone where they eventually meet (results not shown).

These scientific experiments provided a basis for the *Fifty Percent Human* project. Some of the metaphoric questions that were postulated could and can be answered by existing scientific methods, whereas for some, further experimentation and alteration of methods in the laboratory is necessary. For instance, in order to fully understand microbial companionship, a co‐occurrence network analysis would be needed, requiring a larger sample set. Related questions, however, such as the question of changing perceptions of strangeness and otherness**,** were approached from a different angle and are more open to artistic and philosophical approaches: ‘How can an imaginary world facilitate a radically new view on biological rules, hierarchies, dimensions and scales?’

## From science to art

The scientific answers to the questions central to *Fifty Percent Human* formed the starting point to create an in‐between space installation in which people could literally meet their microbes. This in‐between space aimed to create a tangible display of an imaginary world intended to break down hierarchies, dimensions and scales to engage the public in a fascination of the uncertain, the divided, the distorted, the never pure, the ever‐connecting, the swarming, the open, the living and a sense of empathy.

Practically, the PCA plots describing ‘Who is there?’ (Fig. 1A) and ‘How do they communicate?’ (Fig. 1B) were graphically superimposed to serve as a starting point for the floor plan of the imaginative installation (Fig. 1C). The other scientific, artistic and philosophical results served as further inspiration in generating this in‐between space.

*Fifty Percent Human* presents the visitor with a damp environment filled with enlarged transparent and liquid membrane enclosed microbial cells, collectively swimming, lying or floating (Fig. 1D–F, Installation at Zone2Source, Greenhouse Amstelpark, Amsterdam). Visitors were invited to try to reach out and encounter their non‐verbal micro‐companions through touch. The installation challenged visitors to reimagine their body and open up towards enlarged entanglements of microbial life forms, linked to the paradigm shift that we are in essence fifty per cent microbial. The installation intended to democratize complex scientific research and aims to rethink, reshape and deepen our understanding of fundamental aspects of our being‐in‐the‐world. We sought to encourage a OneHealth perspective on microbes as a web connecting the health of humans, animals and plants – all living species and the environment.

## Reflection and reception

### Artist

We could, with good reason, claim to live in a revolutionary time, and the accomplishments of the biosciences will clearly have an enormous impact on the fundamental assumptions, which structure our society, as well as on more applied areas such as medicine. The discourse between art, history, philosophy of science, science and beyond will remain as a crucial necessity to question the development of scientific discoveries.

### Science philosopher

*Fifty Percent Human* felt different from other multidisciplinary projects, as people from the different disciplines were deeply involved in a common thought process and a common creative act. The answers generated in the project are not only relevant to our engagement with the microcosm, but also to our scientific and artistic practices as well as to the way we exchange knowledge, ideas and experiences in the future.

### Scientists

Scientists tend to look at microbes in a reductionist way, by breaking down complex systems into smaller elements in order to make them easier to study. But, by doing so, have they not also lost the intrinsic respect for these living creatures? Do we embrace our microbiome in our daily life – or does it rather stay an abstract object of scientific curiosity? Do we truly acknowledge that being *Fifty Percent Human* also means that we are *Fifty Percent Microbe*? Discussing these questions with artists, science historians and scientists and engaging the wider public provided new avenues of thinking, feeling and sensing microbes as an intrinsic part of our self.

## Conclusion

Scientific language can only be understood by a small scientific community. Hence one way to interpret scientific matters and reach out to the public is via art. Collaborations between multidisciplinary fields such as art, history, philosophy of science and science, such as *Fifty Percent Human*, allow the merging of visions, communication, technical knowledge and methodology, to create positive advancements in society. It is our hope that *Fifty Percent Human* inspires more scientists to explore innovative and unique ways to communicate their science to the broader audience.

## Meeting your microbes

*Fifty Percent Human* has already travelled through Europe with stops in the Netherlands, Austria, the UK and Norway. It continues to be further developed. Find more information about the project on: http://www.sonjabaeumel.at/works/bacteria/fifty-percent-human/ and about upcoming exhibitions, talks and workshops here: http://www.sonjabaeumel.at/info/news.

## Conflict of Interest

None declared.
